# A tracer liquid image velocimetry for multi-layer radial flow in bioreactors

**DOI:** 10.1186/s12938-015-0002-z

**Published:** 2015-02-13

**Authors:** Yu-Bao Gao, Jiu-Xing Liang, Yu-Xi Luo, Jia Yan

**Affiliations:** School of Engineering, Sun Yat-sen University, Guangdong, China

**Keywords:** Bioreactor, Fluid shear stress, Image velocimetry, Computational Fluid Dynamics, Parallel plates flow chamber

## Abstract

**Background:**

This paper presents a Tracer Liquid Image Velocimetry (TLIV) for multi-layer radial flow in bioreactors used for cells cultivation of tissue engineering. The goal of this approach is to use simple devices to get good measuring precision, specialized for the case in which the uniform level of fluid shear stress was required while fluid velocity varied smoothly.

**Methods:**

Compared to the widely used Particles Image Velocimetry (PIV), this method adopted a bit of liquid as tracer, without the need of laser source. Sub-pixel positioning algorithm was used to overcome the adverse effects of the tracer liquid deformation. In addition, a neighborhood smoothing algorithm was used to restrict the measurement perturbation caused by diffusion. Experiments were carried out in a parallel plates flow chamber. And mathematical models of the flow chamber and Computational Fluid Dynamics (CFD) simulation were separately employed to validate the measurement precision of TLIV.

**Results:**

The mean relative error between the simulated and measured data can be less than 2%, while in similar validations using PIV, the error was around 8.8%.

**Conclusions:**

TLIV avoided the contradiction between the particles’ visibility and following performance with tested fluid, which is difficult to overcome in PIV. And TLIV is easier to popularize for its simple experimental condition and low cost.

## Background

In cell cultivations for tissue engineering, the perfusion bioreactors with multi-layer circular parallel plates scaffold were widely used. Ansede, Swift, Wang, Zhang, Lei et al. [1-5] all employed this type of perfusion bioreactors to implement their researches. However, relative few studies have addressed the uniformity of fluid shear stress among different layers. In fact, the magnitude of fluid shear stress significantly affects the proliferation and differentiation of cells [6,7]. And in many cell cultivations, the uniform level of fluid shear stress required was up to 0.001 Pa [8-11]. So it is necessary to determine the real magnitude of fluid shear stress in each layer by measuring the actual velocity distribution.

A widely used measurement method for fluid velocity is Particle Image Velocimetry (PIV) [12]. Jonathan, Bahar, Georgecet, Stephan, et al. [13-16] used PIV to measure flows within bioreactors for cell culture purposes. PIV utilized advanced image acquisition, such as high speed cameras and laser light sources, and complicated computer processing technology to measure the full-field flow. For it imaged in a cross-section illuminated with a sheet light source, it is unavoidable that some particles move into or shift out of the light sheet in measuring process. This change of particles resulted in the errors of algorithms based on correlation in PIV [17]. Moreover, the contradiction between the following performance and visibility of tracer particles is still difficult to overcome in PIV [18]. Reducing the size of particle improves the following performance with the fluid flow, but it weakens the visibility of particle [19]. The typical diameter of particle used in PIV is 3–300 μm. To balance this contradiction, Santiago; Chia et al. employed μ-PIV [20,21]. μ-PIV adopted 100–800 nm diameter micro-particles and utilized microscope to take images. But such small particles significantly affect the accuracy of measurements [21]. And the small imaging region of microscope restricts the magnitude of velocity to only 5 mm/s currently [22]. Furthermore, the concentration of seeding particles maintained around 1% in PIV. It turned the original single-phase flow into solid–liquid two-phase flow. In this situation, the consistency of the particles and flow field is of concern.

In the bioreactor with multi-layer circular parallel plates scaffold, the flow field in every layer is radial flow. In cell cultivations, the goal of measurement is the distribution uniformity of fluid shear stress among different layers, but not the characterization of full flow field. This goal can be achieved by measuring the velocities of fluid among every layer at one vertical line. In a multi-layer cylindrical radial flow field, when the total flow rate keeps constant, the position of the vertical line can be selected arbitrarily. In this research the focus is measurement precision, but not velocity gradient. Therefore, the measuring areas in which fluid velocity varied smoothly can be selected. For such measurement cases, a Tracer Liquid Image Velocimetry (hereinafter referred to as TLIV) was presented. The TLIV simply used general industrial digital cameras and natural light source, adopted liquid to replace particles as tracer. While for the tracer liquid is deformable, poly-dispersed and easy diffused, the accuracy of measurement is necessary to analyze. To eliminate the adverse effects of deformation, a sub-pixel positioning algorithm was utilized. And to overcome the measurement perturbations caused by the diffusion, a neighborhood smoothing algorithm was employed. For the lack of validated mathematical model of circular parallel plates bioreactors, the TLIV experiments were carried out in a rectangular parallel plates flow chamber. And to verify the precision, a mathematical model of the flow chamber and simulations using Computational Fluid Dynamics (CFD) [23] were performed, respectively.

## Methods

### Experimental set-up

**Figure 1:**
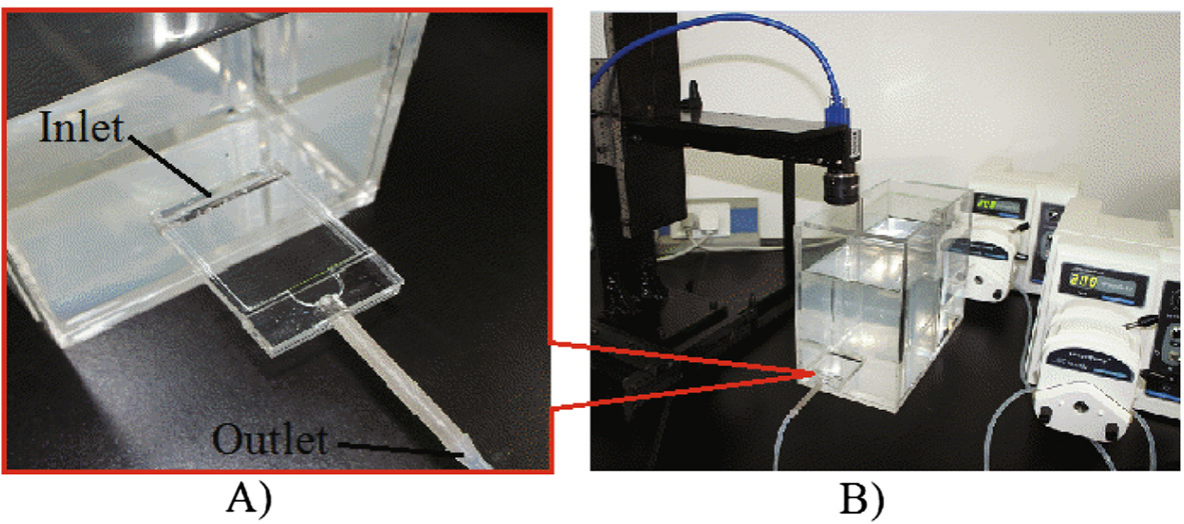
**Experimental set-up. A**: Rectangular parallel plates flow chamber; **B**: Experimental set-up of velocimetry.

The experimental equipment for validating the TLIV was shown as Figure 1. Driven by a peristaltic pump, water flowed from a reservoir (un-shown in Figure 1) into the tank in which a baffle was coupled to avoid affecting the flow field in parallel plates chamber. Then it flowed from inlet to outlet of the chamber driven by another peristaltic pump. Dye tracer was injected at the middle in the inlet through a syringe needle (un-shown in Figure 1) with 0.5 mm aperture. The thickness and width of injected dye tracer should separately be kept within 1.5 mm and 5 mm to avoid adhering to the plates or lateral walls during it flowing through the chamber. A camera was placed right above or on the side of the parallel plates chamber to get images of the dye tracer flowing in the chamber.

### Tracer liquid image velocimetry

- i) Pre-processing

**Figure 2:**
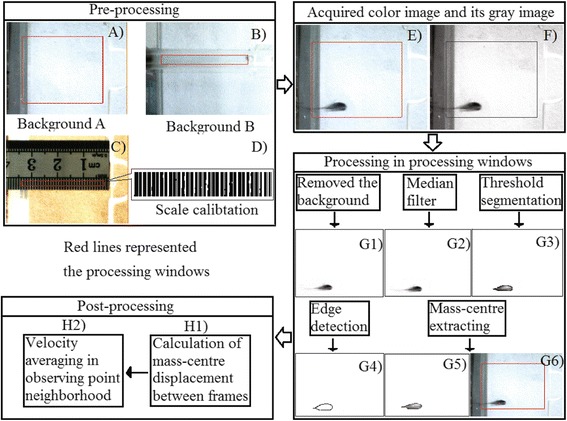
**Tracer Liquid Image Velocimetry (TLIV) schematic. A**: Background image of top view. **B**: Background image of side view. **C**-**D**: Scale calibration. **E**-**F**: Acquired color image and its gray image. **G1**-**6**: Sub-pixel positioning algorithm. **H1**-**2**: Post-processing.

Before injecting tracer liquid, images of flow chamber filled with water were taken as the background images. Figure 2 A and B were the background images separately taken from top and side views. Let *b*(*x*, *y*) represent the intensity of background images, where (*x*, *y*) denote the coordinate of arbitrary pixel.

Then in all images, a rectangular region containing interesting objects was select as the processing windows, shown as the red frames in Figure 2.

For calibrating the scale in images, a graduate ruler was put into the middle layer in the flow chamber. Then the center lines of scales were extracted out. The number of pixels between every two center lines was the conversion coefficient *k* between pixel interval and actual size. Figure 2 C-D illustrates the scale calibration.

- ii) Sub-pixel positioning algorithm

Firstly, the acquired color images were transformed into gray images, notated as *f*(*x*, *y*), as shown in Figure 2 F.

To enhance the interesting objects, a subtraction operation was carried out by using the form

1 $$ {f}_{\mathrm{sub}}\left(x,y\right) = f\left(x,y\right) - b\left(x,y\right) $$

where *b*(*x*, *y*) represents the intensity of background images.

Then a median filter [24] was used to reduce noise. Afterward, to extract the objects, an intensity thresholding algorithm was implemented by the expression

2 $$ g\left(x,y\right)=\left\{\begin{array}{l}1\kern1em  if\ f\hbox{'}\left(x,y\right)>T\\ {}0\kern1em  if\ f\hbox{'}\left(x,y\right)\le T\end{array}\right. $$

where *T* is an intensity threshold and *f* ' (*x*, *y*) is the output images of the median filter.

To determine the boundary, *B*, of interesting object, an edge detection algorithm with Canny edge detector [24] was carried out. Afterward, the coordinate of tracer mass center can be calculated by using the following expression

3 $$ \begin{array}{l}{x}_c=\frac{{\displaystyle \sum {f}_i\left({x}_i,{y}_i\right){x}_i}}{{\displaystyle \sum {f}_i}}\\ {}{y}_c=\frac{{\displaystyle \sum {f}_i\left({x}_i,{y}_i\right){y}_i}}{{\displaystyle \sum {f}_i}}\end{array} $$

where (*x*_*i*_, *y*_*i*_) denote the coordinate of arbitrary pixel *i*, for all *i* ∈ *B*, and *f*_*i*_(*x*_*i*_, *y*_*i*_) denote the intensity of pixel *i*. The Eq. (3) is of sub-pixel algorithm that the coordinate obtained can be less than 1 pixel.

Figure 2 G1-5 illustrates the procedures of the sub-pixel positioning algorithm.

- iii) Post-processing

Let set *A* represent the extracted coordinates of object positions from a set of continual images. Then the instantaneous displacement speed of objects between any two continual image *i* and image *i*-1 can be calculated by

4 $$ {d}_i=\left({a}_i-{a}_{i-1}\right)/\left({t}_i-{t}_{i-1}\right) $$

where *a*_*i*_, *a*_*i* − 1_ ∈ *A* and *t*_*i*_, *t*_*i* − 1_ separately denote the photographing time (in unit of millisecond) of images *i* and *i*-1.

For reducing the perturbations of the displacement, a neighborhood smoothing [24] algorithm was implemented by using following expression

5 $$ \overline{d_i}=\frac{1}{2r+1}{\displaystyle \sum_{i=-r}^r{d}_i} $$

where *r* denote the neighborhood range of object position *i*.

Then the moving velocity of object at *a*_*i*_ can be obtained by

6 $$ {v}_i=\overline{d_i}/k $$

where 1/*k* is the physical distance ( in unit of millimeter) between two neighboring pixels, obtained in pre-processing.

Figure 2 H1-2 illustrates the procedures of post-processing.

According to the kinematic law of mass center in theoretical mechanics [25], in mass system, the movement law of mass center is completely equal with the movement of one mass point. The acceleration of mass center depends on the magnitude and direction of principal vector of the external force system, while is independent of internal force of mass system and the acting position of external force. Though the tracer liquid cannot be rigid, and it will be deformed and diffused in the flow field. If the diffusion exhibits symmetry statistically, the effect of diffusion will be counterbalanced. The time between two continuous images of tracer was less than 60 ms (for actual frame rate ≥ 17 fps). Consequently, for incompressible fluid, if the deformation of tracer liquid profile can be ignored during such short time, the tracer can be considered as rigid body. Then the theory mentioned in reference [25] can be used for tracer liquid. It means that the moving velocity of mass center can represent the tracer velocity. For verifying these assumptions, the validating experiments were carried out in this paper.

### Experimental validations and operating conditions

The parallel plates flow chamber shown as Figure 1 A was 3 mm high, 38 mm wide and 55 mm long. Tested fluid was liquid-water. The rotational speed of the peristaltic pump was 200 rpm and the flow out rate was 57 ml/min. The image processing windows A and B shown in Figure 2 A-B separately were 370 pixels and 50 pixels high. Two windows both were 490 pixels wide, and the distances between each window and the outlet boundary of flow chamber both were 2 mm.

Tracer liquid was water-diluted black ink (Hero$R$) with a density of 0.999 g/cm^3^. The injecting position of tracer located at the horizontal middle layer of inlet. The injecting direction was normal to flowing direction of tested fluid.

Imaging device was industrial digital camera (CatchBEST$R$ MU3C500C) with USB 3.0 interface and frame cache. Imaging background was white baffle illuminated by natural light source. The frame rate was 15 – 20 fps @ 720 × 518 pixels. Images were processed by software MATLAB$R$.

Photographing positions were separately located at top and side of parallel plates flow chamber.

### Comparing validations

To verify the TLIV experiments, mathematical models of the parallel plates flow chamber were utilized. The velocity model is [26]

7 $$ u=\frac{3}{2WH}\left[1-4{\left(\frac{y}{H}\right)}^2\right]{Q}_V $$

where *W*, *H*, *Q*_*V*_, *y* separately represent the width, height, flow rate of flow chamber, and the vertical coordinate. The original point located at the center of inlet. When *y* = 0 mm, the velocity gets its maximum value

8 $$ {u}_{\max }=\frac{3}{2WH}{Q}_V=\frac{3}{2}\overline{u} $$

where *ū* is mean velocity

9 $$ \overline{u}=\frac{Q_V}{WH} $$

The fluid shear stress on parallel plates is

10 $$ \tau =\frac{6\mu }{W{H}^2}{Q}_V $$

where μ is viscous coefficient (this paper μ = 0.001003 kg/m-s).

Moreover, Computational Fluid Dynamics was used to simulate the flow field. The size of simulated geometry was the same as the parallel plates flow chamber. The geometry was meshed in software ICEM (ANSYS$R$) with non-structure grid. The flow field was calculated in software Fluent (ANSYS$R$) with inlet velocity of 0.0083 m/s, steady laminar model ignoring energy transform.

## Results

Separately according to Eq. (9) and Eq. (8), the mean and maximum velocities separately were 8.3 mm/s and 12.5 mm/s at the flow rate of 9.5E-7 m^3^/s. On the base of Eq. (7), for y = −0.5 mm and −0.6 mm, the velocities were 11.11 mm/s and 10.5 mm/s, respectively.

**Figure 3:**
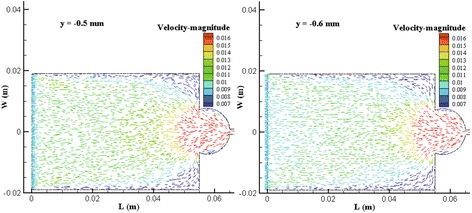
**Velocity vector distribution of CFD.**

By CFD, The flow rate was 9.4996E-7 m^3^/s. And for y = −0.5 mm and −0.6 mm, the distributions of velocity vector were shown as Figure 3.

By the pre-processing shown as Figure 2 C-D, the value of coefficient *k* in Eq. (6) was 15.5 pixel/mm.

**Figure 4:**
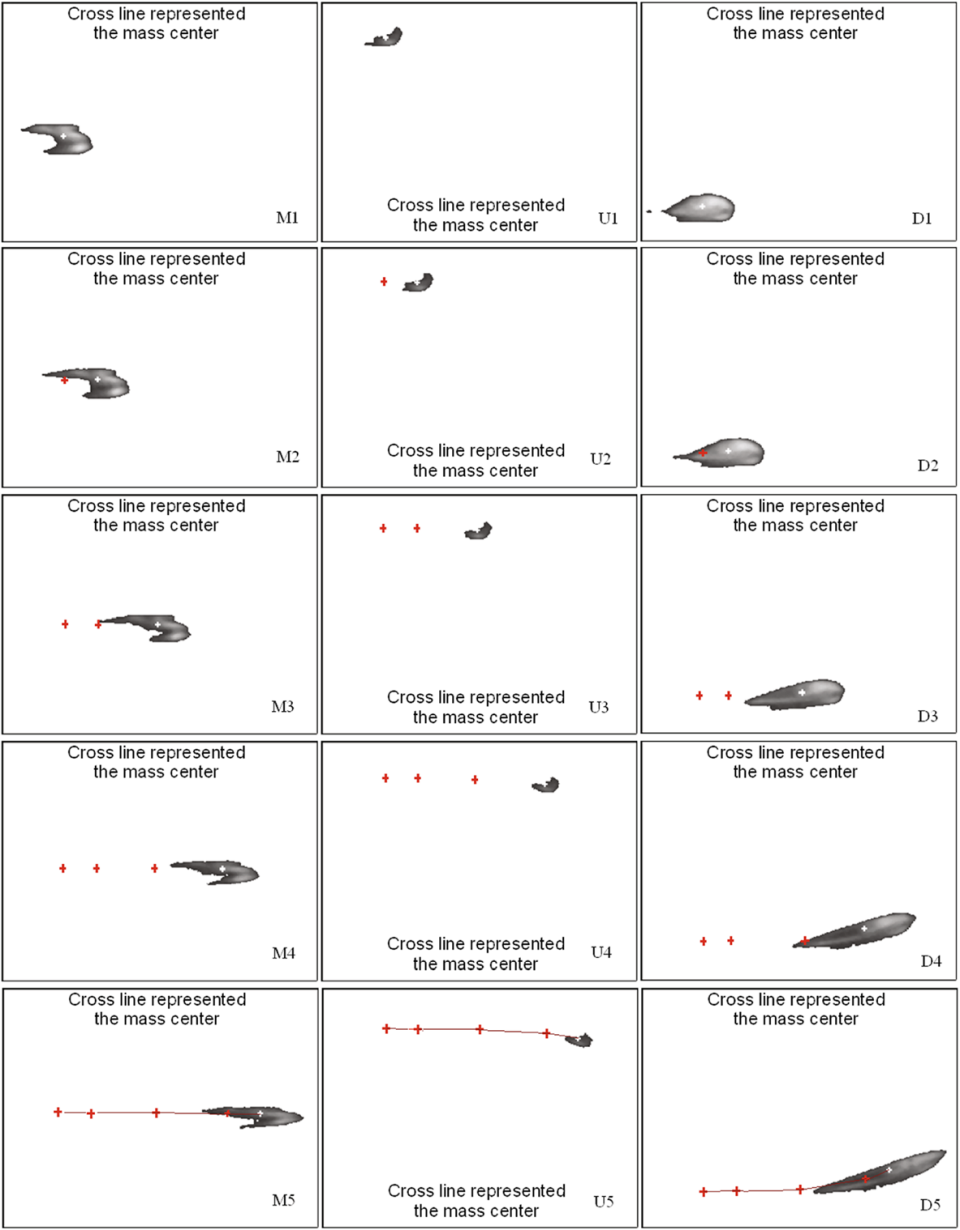
**Gray images in processing windows of top views.** M1 ~ 5: Injection position of tracer near middle area. U1 ~ 5, and D1 ~ 5: Injection position of tracer near two sides areas, respectively. Curves in M5, U5and D5 represented the pathlines of mass center.

The illustrations of TLIV on top view were shown in Figure 4. Its velocity distribution at 5 observing points were shown in Table 1. In Figure 4, M1-5, U1-5 and D1-5 were corresponding to Cases 2, 1, and 3 in Table 1, respectively.

**Table 1:** **Velocity of tracer liquid at 5 observing points by TLIV on top view**

**Cases**	**Position 1**		***V***	**Position 2**		***V***	**Position 3**		***V***	**Position 4**		***V***	**Position 5**		***V***
	***C***_**y**_	***C***_**x**_		***C***_**y**_	***C***_**x**_		***C***_**y**_	***C***_**x**_		***C***_**y**_	***C***_**x**_		***C***_**y**_	***C***_**x**_	
1	56	102	11.4	56	152	11.9	58	245	11.2	69	351	11.5	81	402	12.8
2	192	103	8.5	190	150	9.9	188	244	11.9	186	351	13.4	184	404	14
3	321	98	9.7	319	148	10.3	311	254	10.4	295	352	11.3	284	389	13.7
Mean		101	9.9		150	10.7		248	11.2		351	12.1		398	13.5

*V* was tracer liquid velocity in unit of mm/s. *C*_x_, *C*_y_ separately represented column and row coordinates of tracer liquid, both in unit of pixel.

**Figure 5:**
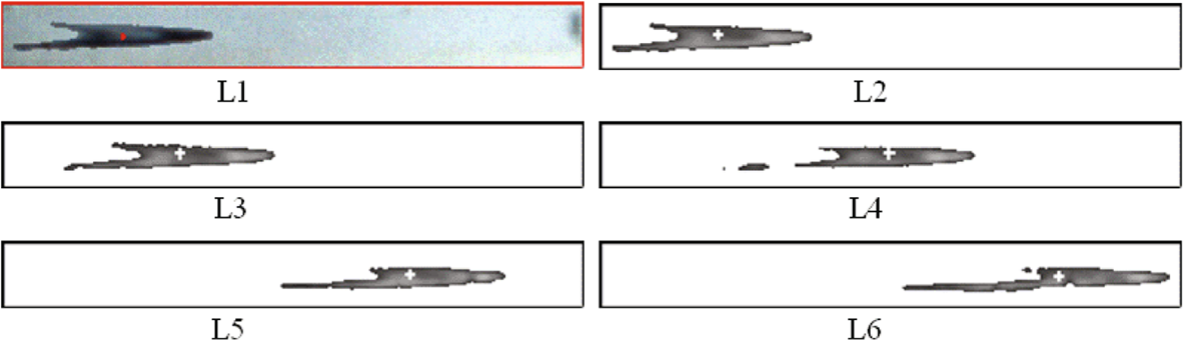
**Images in processing windows of side views.** L1: color image with mass center located at position 1 in Table 2. L2 ~ 6: gray images with mass center separately located at positions 1 ~ 5 in Table 2. Cross lines represented the mass centers.

**Figure 6:**
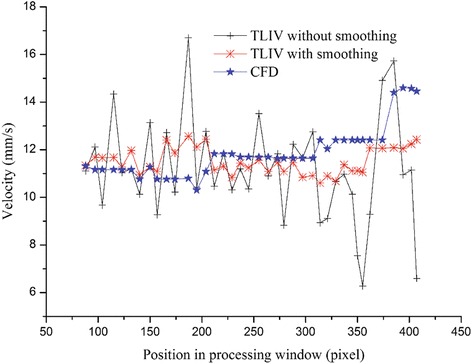
**Velocity distributions.** Black curve: obtained by TLIV without neighborhood smoothing algorithm; Red curve: obtained by TLIV with neighborhood smoothing algorithm; Blue curve: obtained by simulation.

The illustrations of TLIV at side view were shown as Figure 5. Its velocity distributions were shown in Figure 6. For comparing, the simulated data was also plot in Figure 6.

For 3 trial cases of TLIV at side view, the velocities at 5 observing points were shown in Table 2. Figure 5 was corresponding to the Case 1 in Table 2.

**Table 2:** **Velocity of tracer liquid at 5 observing points by TLIV at side view**

**Cases**	**Position 1**		***V***	**Position 2**		***V***	**Position 3**		***V***	**Position 4**		***V***	**Position 5**		***V***
	***C***_**y**_	***C***_**x**_		***C***_**y**_	***C***_**x**_		***C***_**y**_	***C***_**x**_		***C***_**y**_	***C***_**x**_		***C***_**y**_	***C***_**x**_	
1	30.6	97	10.9	30.7	150	11.1	31.0	246	11.2	32.1	351	11.5	32.2	403	12.2
2	30.3	102	10.5	30.7	153	11.2	30.6	248	11.5	32.1	348	11.6	33.0	392	11.8
3	31.0	101	11.3	31.1	150	11.5	31.5	252	11.5	32.4	347	11.6	32.7	388	11.7
Mean	30.8	100	10.9	30.8	151	11.3	31.0	249	11.4	32.2	349	11.6	32.6	394	11.8

*V* was tracer liquid velocity in unit of mm/s. *C*_x_, *C*_y_ separately represented column and row coordinates of tracer liquid, both in unit of pixel.

The variable *C*_y_ shown in Table 2 can be transformed to *y* by following expression

11 $$ y=\left(15.5\times 3/2\hbox{-} {C}_{\mathrm{y}}\right)/15.5 $$

where 3 represented the height (in unit of mm) of the chamber, multiplying by 15.5 to transform its unit of mm into pixel. Then Eq. (11) indicated the calculation of the vertical distance (in unit of mm) between the position descripted by *C*_y_ and the horizontal middle layer in the chamber.

Substituting the values of *y* into Eq. (7), the model-based velocity, *V*_C_, can be calculated. The errors between *V* shown in Table 2 and *V*_C_ can be obtained and were shown as Table 3.

**Table 3:** **Errors between TLIV and mathematical model**

**Cases**	**Position 1**		**Position 2**		**Position 3**		**Position 4**		**Position 5**	
	***E***_**V**_	***RE***_**V**_	***E***_**V**_	***RE***_**V**_	***E***_**V**_	***RE***_**V**_	***E***_**V**_	***RE***_**V**_	***E***_**V**_	***RE***_**V**_
1	0.38	3.3	0.12	1.1	0.09	0.8	0.81	7.5	1.57	14.8
2	0.88	7.7	0.02	0.2	0.23	2	0.91	8.5	1.51	14.6
3	0.19	1.7	0.39	3.5	0.56	5.1	1.03	9.8	1.27	12.1
Mean	0.35	3.1	0.12	1	0.29	2.6	0.95	8.9	1.45	13.8

*E*_V_ = |*V* − *V*_C_|, denote absolute error. *RE*_V_ = *E*_V_/*V*_C_ × 100 %, denote relative error.

The variable *C*_x_ can be transformed to *L* shown in Figure 3 by expression

12 $$ L=\left[55-\left(490/15.5+2\right)\right]+{C}_x/15.5 $$

where 55 was the length (in unit of mm) of the chamber. 490 was the length (in unit of pixel) of processing window. And 2 was the distance (in unit of mm) between the left side of processing window and the left side of the chamber. Then Eq. (12) indicated the calculation of the horizontal distance (in unit of mm) between the position descripted by *C*_x_ and the inlet of the chamber.

Based on Eqs. (11) and (12), the coordinate (*C*_x_, *C*_y_) in image processing window can be transformed to the coordinate of (*L*, *y*) shown in Figure 3 where *y* indicated the vertical height in flow chamber. Then the simulated velocity of flow field corresponding to (*C*_x_, *C*_y_) can be collected from simulated results through indexing (*L*, *y*).

As a result, combining the values of *y*, *L*, and *W* = 0, the simulation-based velocity, *V*_CFD_, can be collected. Then the errors between *V* and *V*_CFD_ can be obtained and were shown as Table 4.

**Table 4:** **Errors between TLIV and CFD**

**Cases**	**Position 1**		**Position 2**		**Position 3**		**Position 4**		**Position 5**	
	***E***_**V**_	***RE***_**V**_	***E***_**V**_	***RE***_**V**_	***E***_**V**_	***RE***_**V**_	***E***_**V**_	***RE***_**V**_	***E***_**V**_	***RE***_**V**_
1	0.2	1.8	0	0	0.5	4.2	1.1	8.7	2.1	14.6
2	0.8	7.1	0.1	0.9	0.3	2.5	1.2	9.4	2.3	16.1
3	0.4	3.7	0.5	4.5	0.3	2.6	0.9	7.2	2.3	16.4
Mean	0.2	1.8	0.2	1.8	0.2	1.5	1	7.9	2.2	15.7

*E*_V_ = |*V* − *V*_CFD_|, denote absolute error. *RE*_V_ = *E*_V_/*V*_CFD_ × 100 %, denote relative error.

## Discussion

According to Eq. (7), the velocity distribution is independent of fluid viscosity. So in this paper the water was used for validation.

In Figure 3, the fluid near outlet flowed from two sides toward middle. This phenomenon was caused by the stagnant flow in two corners. The pathlines of mass center shown in Figure 4 U5 and D5 exhibited the same result. Thus the top view of TLIV reflected the stagnant flow existing in the flow chamber.

For the stagnant, the actual width of flow field narrowed as fluid flowed to outlet. When total flow rate keeps constant, velocity increases as the width of flow field decreases. Figure 3 and Tables 1 and 2 indicated that the simulated and measured results both exhibited this trend. But Eq. (7) is based on the assumption that the length of flow chamber is infinite. It can’t reflect horizontal velocity variation.

In Table 1, some values deviated the mean velocity more than 1 mm/s. These conspicuous errors were resulted by the sharp change of background illuminant shown as Figure 2 A. While in Figure 2 B the illumination of background image was smoother. So in Table 2, at each observing point, the errors between every velocity and the mean velocity were less than 0.4 mm/s. At side views of the flow chamber, for the light diffused through a longer path, sharp illuminant changes were avoided. Thus Good performance of TLIV can be achieved under natural light source. The following discussions were mainly based on side views.

In all experiments, tracer was injected at horizontal middle layer of inlet. For the density of tracer is close to which of water, the vertical height of tracer can keep steady in flow chamber. The values of *C*_y_ in Table 2 meet this characteristic. Its variation was less than 3 pixels.

In Figures 4 and 5, the deformations of tracer liquid were obvious. Such as from M1 to M5 in Figure 4, the profile of tracer varied toward lengthening. From Eq. (7) it can be seen that the velocities of flow in different position within the chamber exist discrepancy, which resulted in the obvious deformations of tracer. But from their mass centers extracted in Tables 1 and 2, it can be seen that the sub-pixel positioning algorithm can eliminate the adverse effects of deformations.

A noticeable effect on object positioning is liquid diffusion, shown as the black curve in Figure 6. The red curve in Figure 6 demonstrated that the neighborhood smoothing algorithm can effectively restrict such adverse effects. However, the smoothing process simultaneously filtered the sharp changing components of velocity. As shown in Figure 6, at the positions of column = 170, 370, and 400 pixels, though the simulated values changed obviously by the variation of *C*_y_, the measured values didn’t follow these abrupt changes. This discrepancy was resulted by the influence of smoothing algorithm. For such sharply varied velocity, the maximum relative error between measured and simulated values was up to 16.4%, shown as positions 4–5 in Table 4. But as mentioned before, the suitable positions for measurement using TLIV can be selected in the region where the velocity keeps smooth, such as positions 1–3 shown in Table 2. In these areas, the mean relative error range between measured and simulated data was [1.5%,1.8%]. While in similar validations by PIV, the error was around 8.8% [27,28].

According to Eq. (7) and Eq. (10), the fluid shear stress is proportion to velocity. Therefore, the distribution of fluid shear stress can be determined by the measurement of velocity.

For the dilution effects of water on the tracer liquid, the TLIV is not applicable to the situations that need to trace over a long period of time. All tests in this paper were no more than 5 seconds. In next research, we will find a tracer liquid that can’t be dissolved by water.

Though there is discrepancy between the flow field in rectangular flow chamber and that in circular parallel plates bioreactors, the main purpose of this paper is to validate the feasibility and measuring precision of TLIV. In future study, we will establish mathematical model for circular parallel plates bioreactors, and experimentally verify it with the TLIV.

## Conclusions

In cell cultivations, it is necessary to measure the velocity distribution of fluid in bioreactors. For a cylindrical multi-layer radial flow field, we can select the measuring regions in which velocities varied smoothly to determine the distribution uniformity. To get good measuring precision by simple devices, this paper provides a velocimetry. It used tracer liquid to replace particles, and employed digital image processing technology to solve the problems caused by the new tracer. Its measuring precision sufficiently met the requirement of application.

## Abbreviations

CFD: Computational Fluid Dynamics
PIV: Particles Image Velocimetry
TLIV: Tracer Liquid Image Velocimetry
